# Proteome Analysis and Serological Characterization of Surface-Exposed Proteins of *Rickettsia heilongjiangensis*

**DOI:** 10.1371/journal.pone.0070440

**Published:** 2013-07-23

**Authors:** Yong Qi, Xiaolu Xiong, Xile Wang, Changsong Duan, Yinjun Jia, Jun Jiao, Wenping Gong, Bohai Wen

**Affiliations:** State Key Laboratory of Pathogen and Biosecurity, Beijing Institute of Microbiology and Epidemiology, Beijing, China; University of Texas Medical Branch, United States of America

## Abstract

**Background:**

*Rickettsia heilongjiangensis*, the agent of Far-Eastern spotted fever (FESF), is an obligate intracellular bacterium. The surface-exposed proteins (SEPs) of rickettsiae are involved in rickettsial adherence to and invasion of host cells, intracellular bacterial growth, and/or interaction with immune cells. They are also potential molecular candidates for the development of diagnostic reagents and vaccines against rickettsiosis.

**Methods:**

*R. heilongjiangensis* SEPs were identified by biotin-streptavidin affinity purification and 2D electrophoreses coupled with ESI-MS/MS. Recombinant SEPs were probed with various sera to analyze their serological characteristics using a protein microarray and an enzyme-linked immune sorbent assay (ELISA).

**Results:**

Twenty-five SEPs were identified, most of which were predicted to reside on the surface of *R. heilongjiangensis* cells. Bioinformatics analysis suggests that these proteins could be involved in bacterial pathogenesis. Eleven of the 25 SEPs were recognized as major seroreactive antigens by sera from *R. heilongjiangensis*-infected mice and FESF patients. Among the major seroreactive SEPs, microarray assays and/or ELISAs revealed that GroEL, OmpA-2, OmpB-3, PrsA, RplY, RpsB, SurA and YbgF had modest sensitivity and specificity for recognizing *R. heilongjiangensis* infection and/or spotted fever.

**Conclusions:**

Many of the SEPs identified herein have potentially important roles in *R. heilongjiangensis* pathogenicity. Some of them have potential as serodiagnostic antigens or as subunit vaccine antigens against the disease.

## Introduction

*Rickettsia heilongjiangensis* is an obligate intracellular Gram-negative bacterium that belongs to the tick-borne spotted fever group (SFG) of rickettsiae [1]. It was first isolated in 1983 from *Dermacentor silvarum* ticks in Suifenhe, in the Heilongjiang Province of China [2]. *R. heilongjiangensis*, which is pathogenic to humans [3]–[7], causes the disease now formally known as Far-Eastern spotted fever (FESF). This disease has been diagnosed in patients in Northeastern China [3], Siberia and the Far East of Russia [4], [5], [7], and Japan [6]. After an incubation period of 4 to 7 days, most of the patients naturally infected with *R. heilongjiangensis* experience fever, chills, headache, dizziness, myalgia, arthralgia and anorexia, after which most of the patients show signs of a macular or maculopapular rash, and some of the patients have a primary lesion (otherwise called an eschar) that appears at the site of tick attachment, as well as lymphadenopathy close to the eschar itself [5]. Almost half of the patients have hepatomegaly accompanied with increased alanine aminotransferase and/or aspartate aminotransferase activity, indicating that *R. heilongjiangensis* infection in these patients causes liver lesions [5]. It has been suggested that FESF is an important emerging infectious disease in Northeast Asia. Our previous study using a mouse model revealed that *R. heilongjiangensis* caused severe systemic infection and that the pathological lesions in the infected organs (lungs, spleen, and brain) were associated with host inflammatory responses induced by *R. heilongjiangensis* [8].

Like other pathogenic SFG rickettsiae, *R. heilongjiangensis* has the ability to invade and proliferate within vascular endothelial cells and cause cell injury and death [9]. A “zipper-like” invasion strategy has been proposed for rickettsia invasion of non-phagocytic host cells [10], [11]. Zipper-like invasion is a receptor-mediated invasion mechanism, whereby a bacterial protein induces host intracellular signaling through extracellular stimulation of a membrane receptor [12], which suggests that rickettsiae surface-exposed proteins (SEPs) play a fundamental role in host-rickettsial interactions.

The Sca (surface cell antigen) family proteins of SFG rickettsiae are recognized as the dominant SEPs [12], [13] that play important roles in rickettsial pathogenesis. Sca0 (outer membrane protein A, OmpA) and Sca1 are both involved in attachment of rickettsiae to host cells [14], [15], whereas Sca5 (OmpB) is associated with rickettsiae entry into host cells [16]–[18]. Sca2 functions as a formin mimic that is responsible for the actin-based motility of rickettsiae in host cells [19], [20]. Sca4 activates vinculin and interacts with the actin cytoskeleton of host cells [21]. In addition, OmpA and OmpB are known to be important protective antigens in SFG rickettsiae, and have the ability to efficiently induce humoral and cellular immunity against spotted fever [22].

Rickettsial surface components probably play roles in pathogenicity, such as adherence to and invasion of host cells, intracellular parasite growth, and/or interactions with immune cells. Proteomics analysis of rickettsiae surface molecules has the potential to discover novel molecules involved in bacterial pathogenesis, including those required for invasion of host cells, and those required for efficient induction of specific immune responses against rickettsial infection. Such studies also have the potential to deliver reagents for serological diagnosis of rickettsiosis. In the present study, *R. heilongjiangensis* SEPs were identified by a proteome analysis of its cell surface proteins. Based on these results, recombinant SEPs from *R. heilongjiangensis* were prepared and serologically characterized by protein microarray assays and an enzyme-linked immune sorbent assay (ELISA) using sera from FESF patients or from mice experimentally infected with *R. heilongjiangensis* as well as other rickettsial pathogens.

## Materials and Methods

### Ethics Statement

Specific pathogen-free male C3H/HeN or BALB/c mice (5 to 6 weeks of age) were purchased from the Laboratory Animal Center of Beijing, China. The animal experiments were approved by the Beijing Administrative Committee for Laboratory Animals and the animal care met the standard of the committee. Mice were well cared for during their stay in the facility and all efforts were made to minimize suffering.

Research using samples from humans was approved by the Institutional Review Board of the Beijing Institute of Microbiology and Epidemiology. The study was performed after the receipt of informed written consent from the patients and healthy donors, or their guardians. The data analysis was performed anonymously.

### Mouse and Human Sera

Serum samples from BALB/c mice infected with *R. heilongjiangensis* (054 strain) were prepared as described previously [8]. Sera from BALB/c mice experimentally infected with *R. sibirica* (246 strain), *R. rickettsii* (Sheila Smith), *R. prowazekii* (Madrid E), *R. typhi* (Wilmington), *Orientia tsutsugamushi* (Karp), or *Coxiella burnetii* (Xinqiao) were obtained from the laboratories in our Institute. All the mouse sera used were collected on day 28 post-infection. The titers of specific IgG antibodies in the sera samples were determined through use of their corresponding rickettsial antigens and indirect immunofluorescence assays (IFA) [23].

Nine sera from FESF patients and seven sera from healthy blood donors were obtained from hospitals in northeast China. The serum samples were collected in the hospitals as part of their routine management of patients or blood donors (i.e., it was done without any additional sampling), and data from the patients and blood donors were anonymized. The IgG antibody titers against *R. heilongjiangensis* in the human sera were determined by IFA [23].

Each serum sample was diluted 1∶100 in PBS (8.1 mM Na_2_HPO_4_, 1.9 mM NaH_2_PO_4_, 154 mM NaCl; pH 7.4) and then neutralized overnight using an *Escherichia coli* cell lysate at a final protein concentration of 5 mg/ml before use.

### Cultivation and Purification of *R. heilongjiangensis*

The *R. heilongjiangensis* (054 strain) was propagated in Vero cells (ATCC) and purified by renografin density centrifugation [8]. The viability of newly purified bacteria was ∼95%, as measured using LIVE/DEAD BacLight Bacterial Viability Kits (Invitrogen, Carlsbad, CA) and the numbers of bacteria purified were estimated by quantitative polymerase chain reaction (qPCR) as described previously [8].

### Isolation and Separation of Surface-exposed Proteins

Newly purified *R. heilongjiangensis* (4×10^11^) bacteria were labeled with Sulfo-NHS-SS-Biotin (Thermo Scientific, Rockford, IL) and the biotinylated proteins were captured by streptavidin agarose resin (Thermo Scientific), which was performed as described previously [24], [25]. The captured proteins were eluted from the streptavidin resin with 500 mM dithiothreitol, and the eluted proteins were precipitated using a 2D-Cleanup Kit (GE healthcare, Waukesha, WI). The isolated proteins dissolved in rehydration buffer (7 M urea, 2 M thiourea, 4% CHAPS) were subjected to two-dimensional electrophoresis (2D-PAGE) as described previously [26].

### Identification of Surface-exposed Proteins by Mass Spectrometry

The protein spots on the silver-stained gel were excised and then subjected to in-gel digestion with trypsin [26]. The hydrolysates were analyzed by electrospray ionization tandem mass spectrometry (ESI-MS/MS) and the resultant peptides were mass fingerprinted and compared against the National Center for Biotechnology Information (NCBI) nonredundant databases using the Mascot search engine (http://www.matrixscience.co.uk) [26]. N-terminal signal peptides and non-classical secretion signals in the proteins were predicted by the SignalP 3.0/LipoP 1.0 [27], [28] and SecretomeP 2.0 [29] servers, respectively. SignalP is able to predict the presence and location of N-terminal signal peptides based on a combination of artificial neural networks (NN) and hidden Markov models (HMM) [27]. LipoP 1.0 was used to distinguish lipoproteins, SpaseI-cleaved proteins, and N-terminal membrane helices [28]. SecretomeP, which integrates various posttranslational and localizational aspects of the protein from a large number of other feature prediction servers into the final secretion prediction, is able to predict non-classically secreted proteins [29]. The subcellular location (SCL) of each protein was predicted by the PSORTb 3.0.2 [30] and SOSUI-GramN [31] servers. PSORTb combines several analytical methods [30] and SOSUI-GramN uses the physicochemical parameters of the N- and C-terminal signal sequences, and the total sequence of a protein [31] to predict the SCL of proteins. Both of these software packages generate prediction results for five major cellular and subcellular locations (i.e., cytoplasmic, inner membrane, periplasmic, outer membrane and extracellular) for Gram-negative bacteria. Sequence homology for each of the proteins identified herein was analyzed against proteins from other species of bacteria (available in the NCBI public database) using the Basic Local Alignment Search Tool (BLAST) (http://www.ncbi.nlm.nih.gov/BLAST/). The proteins identified were classified into the Clusters of Orthologous Groups of proteins (COGs) and functions were assigned by the COGnitor server available at NCBI.

### Preparation of Recombinant Surface-exposed Proteins

The genes encoding SEPs were obtained from the genomic sequence of *R. heilongjiangensis* (GenBank accession number: CP002912) [32] and were amplified by the polymerase chain reaction (PCR) with their corresponding primer pairs (Table S1). The amplified genes were expressed in *E. coli* and the expressed recombinant proteins were purified with Ni-NTA affinity resin as described previously [23].

### Surface Localization of YbgF and PrsA by Immunofluorescence Assay

Immune sera against YbgF or PrsA proteins were prepared in C3H/HeN mice. Briefly, each mouse was subcutaneously administered 30 µg of recombinant YbgF or PrsA mixed with Freund’s complete adjuvant (Sigma-Aldrich, St Louis, MO) for the primary immunization, followed by two booster immunizations with 20 µg of the homologous protein mixed with incomplete Freund’s adjuvant (Sigma-Aldrich) on days 28 and 42 post primary immunization. Fourteen days after the last booster immunization, blood samples were collected from the five mice immunized with YbgF or PrsA and the separated sera were pooled together. Sera from mice immunized with *R. heilongjiangensis* whole-cell antigen (WCA) plus adjuvant and adjuvant alone were used as positive and negative controls, respectively. *R. heilongjiangensis* cells smeared onto slides were fixed with ice-cold acetone for 10 min and then incubated with the immune sera (diluted 1∶10 in PBS) for 45 min at 37°C [23]. After three washes with PBS, the rickettsial cells on the slides were incubated with a 1∶200 dilution of Dylight 488-conjugated goat anti-mouse IgG (Thermo Scientific) for 45 min at 37°C. After another three washes, the rickettsial cells on the slides were observed under a fluorescence microscope (Olympus BX60).

### Fabrication of Protein Microarrays

Each of the purified recombinant SEPs was diluted to a final concentration of 300 µg per ml and printed onto polymer slides (Capitalbio, Beijing, China) as described previously [26]. Each protein was printed as five replicate spots, with mouse or human IgG printed as positive controls, while *E. coli* lysates from cells transformed with PET-32a plasmids were used as negative controls [26]. For quality control, the microarray slides were incubated with Cy5-labeled mouse anti-His tag IgG (SBA, Birmingham, AL) and scanned for their fluorescence intensity (FI) and the scanned images were analyzed by GenePix Pro 6.0 software (Molecular Devices, Sunnyvale, CA) [26]. Proteins with a signal-to-background ratio over 3.0 were used for further analysis [26].

### Serological Analysis of Surface-exposed Proteins using Microarray Assays

Recombinant SEPs on the microarray slide were analyzed using various sera according to previous descriptions [26]. Briefly, the microarray slide was blocked with PBS-BSA (PBS, 1% [w/v] BSA, pH 7.4) for 1 h, after which it was incubated with a 100 µl volume of each serum sample at room temperature for 1 h. After incubation, the microarray slide was washed six times in PBST (PBS, 0.05% [v/v] Tween 20, pH 7.4) for 5 min each time on a shaker. The microarray slide was then incubated with goat-anti-mouse IgG-Cy5 or goat-anti-human IgG-Cy5 (SBA) at a 1∶500 dilution at room temperature for 1 h. Following an additional six washes in PBST, the air-dried microarray slide was scanned with a GenePix Personal 4100A scanner (Molecular Devices) and the scanned images were analyzed by GenePix Pro 6.0 (Molecular Devices). The FI value of each protein was calculated by averaging the FI values of five replicate spots in which the backgrounds had been subtracted. The reaction was considered positive if the average FI value of any protein that had been probed with any of the serum samples from infected mice was higher than 3 standard deviations (SD) above the average FI value of the same protein that had been probed with normal mouse sera [33]. In addition, the reaction was considered positive if the average FI value of any protein probed with any of the sera from patients was higher than 2 SD above the average FI value of the same protein probed with sera from healthy people [26]. FI values for proteins probed with sera from infected individuals and control sera from uninfected individuals were analyzed by the Wilcoxon Two-Sample test, whereas FI values for proteins probed with sera from different bacterial infections were performed by the Kruskal-Wallis test, followed by the Student-Newman-Keuls (SNK) test using software SAS 9.1 (SAS Institute, Cary, NC).

### Serological Analysis of Major Surface-exposed Proteins using ELISA

ELISAs were performed as described previously [34]. Briefly, each well of a 96-well microplate (Corning, Corning, NY) was coated overnight at 4°C with 100 µl of each of the purified recombinant proteins at 2.5 µg per ml in ELISA/ELISPOT Coating Buffer (eBioscience, San Diego, CA). Unbound sites in each well were blocked with 200 µl of 1×ELISA Diluent Solution (eBioscience) for 2 h at 37°C. After three washes with PBST, each serum sample was dispensed into three replicate wells (100 µl per well) and the plates were incubated for another hour at 37°C. After another three washes with PBST, 100 µl of a 1∶5000 dilution of horseradish peroxidase (HRP)-conjugated goat anti-mouse IgG (SBA) was added to each well. The plates were incubated at 37°C for another hour and 100 µl of 1×TMB ELISA Substrate Solution (eBioscience) was added to each well for 5 min at room temperature. Thereafter, 50 µl of H_2_SO_4_ (2 M) was added to stop the reaction. The optical density (OD) of each well was read at 450 nm using a microplate reader (UVM 340, ASYS HitechGmbH, Eugendorf, Austria) and the mean OD_450_ of three replicate wells was calculated. The cut-off value of each protein was determined as the mean OD_450_ of normal mouse sera plus 4 SD [35].

## Results

### Identification of Surface-exposed Proteins by ESI-MS/MS Analysis

**Figure 1:**
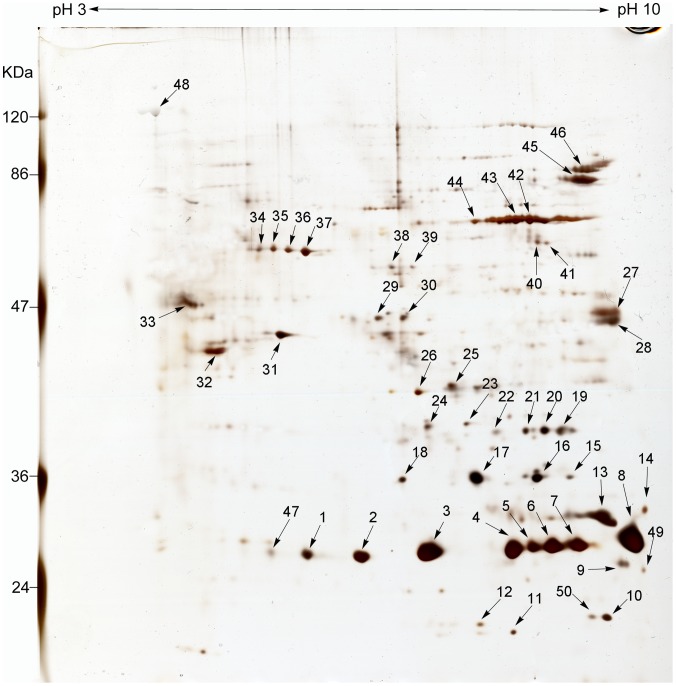
2-D PAGE reference map of surface-exposed proteins in *R. heilongjiangensis*. *R. heilongjiangensis* biotinylated proteins isolated by biotin-streptavidin affinity purification were separated using a pH 3–10 NL IPG strip (Bio-Rad, Richmond, CA) in the first dimension followed by 12% SDS-PAGE. Numbers with arrowheads refer to the protein spots in the silver-stained gel. Protein spots excised from the gel were digested and subjected to ESI-MS/MS analysis. The relative molecular masses of the marker proteins are indicated in kDa on the left side of the figure and the protein spots identified by ESI-MS/MS analysis are listed in Table S2.

The biotinylated SEPs of *R. heilongjiangensis* were isolated by biotin-streptavidin affinity chromatography and these proteins were separated by 2D-PAGE. Approximately 50 protein spots with isoelectric points (pI) ranging from 5 to 10 and molecular masses ranging from 20 to 120 kDa were visualized on the 2D-PAGE gel stained with silver (Figure 1).

ESI-MS/MS analysis identified 25 proteins among 50 protein spots (Figure 1 and Table S2). Most of the 50 spots were identifiable as single proteins. Some spots (such as spots 34 to 37) that appeared as a chain of spots with slightly different pI values were identified as being from the same protein, while a few of the spots (such as spots 7 to 11) contained several different proteins (Figure 1 and Table S2).

Fourteen of the 25 proteins were predicted by the SignalP and SecretomeP servers as classical and/or non-classical secretion proteins. All proteins except Omp1 that were identified as classical secretion proteins were predicted by the LipoP server to be SPaseI-cleaved proteins (Table 1).

**Table 1:** COGs classification and bioinformatics analysis of *R. heilongjiangensis* surface-exposed proteins identified by ESI-MS/MS.

COGs and Protein annotation	Locus Tag	Gene Symbol	Signal peptide^a^ (SignalP/LipoP)	Subcellular Location^b^ (PSORTb/SOSUI-GramN)	Report^c^
M: Cell wall/membrane/envelope biogenesis					
hypothetical protein Rh054_06965	Rh054_06965^d^	*adr1*	Yes/SpI	EC/OM	1, 2, 4
hypothetical protein Rh054_06970	Rh054_06970^d^	*adr2*	Yes/SpI	Non-CYT/OM	1, 2, 3
putative nucleoside-diphosphate sugar epimerase CapD	Rh054_02635	*capD*	No/No	CYT/CYT	
outer membrane protein omp1	Rh054_01180	*omp1*	Yes/No	OM/OM	2, 3, 5, 6
190-kDa cell surface antigen	Rh054_06925^d^	*ompA*	Yes/SpI	OM/OM	3
outer membrane protein B	Rh054_06005^d^	*ompB*	Yes/SpI	OM/EC	1, 2, 3, 4
OmpW family outer-membrane protein	Rh054_00645^d^	*ompW*	Yes/SpI	Unknown/OM	2
dTDP-4-dehydrorhamnose reductase	Rh054_02630	*rfbD*	No/No	CYT/CYT	
cell surface antigen	Rh054_00115^d^	*sca1*	Yes/SpI	OM/EC	1
O: Posttranslational modification, protein turnover, chaperones					
heat shock protease	Rh054_01375	*degQ*	Yes/SpI	PP/EC	1, 6
molecular chaperone GroEL	Rh054_05320	*groEL*	No/No	CYT/CYT	1, 2, 3, 4, 5, 6
Protein export protein prsA	Rh054_04865^d^	*prsA*	Yes/SpI	OM/CYT	1, 2, 3, 4
hypothetical protein Rh054_05640	Rh054_05640	*surA*	Yes/SpI	Non-CYT/OM	
thioredoxin peroxidase 1	Rh054_02600	*tdpX1*	No/No	CYT/CYT	1, 2, 3
J: Translation, ribosomal structure and biogenesis					
50S ribosomal protein L1	Rh054_01050	*rplA*	No/No	CYT/Unknown	
50S ribosomal protein L25/general stress protein Ctc	Rh054_05115	*rplY*	No/No	CYT/CYT	
30S ribosomal protein S2	Rh054_00685	*rpsB*	No/No	CYT/EC	2
Elongation factor Tu	Rh054_05545	*tuf*	No/No	CYT/PP	1, 2, 3, 4
C: Energy production and conversion					
F0F1 ATP synthase subunit beta	Rh054_06725	*atpD*	No/No	CYT, IM/CYT	1, 2, 3, 4, 6
succinate dehydrogenase iron-sulfur subunit	Rh054_00425	*sdhB*	No/No	IM/CYT	
H: Coenzyme transport and metabolism					
bifunctional 5,10-methylene-tetrahydrofolate dehydrogenase/cyclohydrolase	Rh054_03580	*folD*	No/No	CYT/Unknown	
Q: Secondary metabolites biosynthesis, transport and catabolism					
hypothetical protein Rh054_06655	Rh054_06655^d^		No/No	CYT/CYT	4
S: Uncharacterized BCR					
Tol system periplasmic component	Rh054_01780^d^	*ybgf*	Yes/SpI	Non-CYT/CYT	4
Not in COGs					
hypothetical protein Rh054_00610	Rh054_00610^d^		Yes/SpI	OM/EC	1, 3, 5, 6
hypothetical protein Rh054_02285	Rh054_02285		Yes/SpI	Non-CYT/IM	

a Signal peptides and signal peptide types for all of the proteins were predicted with SignalP 3.0 or LipoP 1.0 software available online (http://www.cbs.dtu.dk/services/SignalP-3.0 and http://www.cbs.dtu.dk/services/LipoP-1.0. The websites were accessed on 22 January, 2013).

b The subcellular location of each protein was predicted with PSORTb 3.0.2 or SOSUI-GramN (http://www.psort.org/psortb/index.html and http://bp.nuap.nagoya-u.ac.jp/sosui/sosuigramn/sosuigramn_submit.html. The websites were accessed on 22 January, 2013).

c Proteins with homology to some *R. heilongjiangensis* surface-exposed proteins were also identified on the surfaces or in membrane extracts of some other rickettsiae. 1, 2, 3, 4, 5, and 6 refer to *R. conorii* [15], [36], [37], *R. felis* [38], *R. parkeri* [39], *R. typhi* [40], *Anaplasma phagocytophilum* [24], and *Ehrlichia chaffeensis* [25], respectively.

d Non-classically secreted proteins were predicted with SecretomeP 2.0 (http://www.cbs.dtu.dk/services/SecretomeP/. The website was accessed on 22 January, 2013).

SpI: signal peptide (signal peptidase I); EC: Extracellular; OM: Outer membrane; PP: Periplasmic; IM: Inner membrane. CYT: Cytoplasmic; Non-CYT: Non-Cytoplasmic; BCR: Bacterial conserved region.

Twelve proteins were predicted by PSORTb and/or SOSUI-GramN to localize within the outer membrane and/or extracellular space and five proteins were predicted to reside in the inner membrane, periplasm or other non-cytoplasmic location (Table 1).

In the database search for COGs, the 25 SEPs were classified into eight categories; nine SEPs, including OmpA, OmpB and Sca1 [12]–[15], [17], [18], [40] were classified as belonging to group M, which is predicted to have functions involved in cell wall, membrane or envelope biogenesis (Table 1).

**Figure 2:**
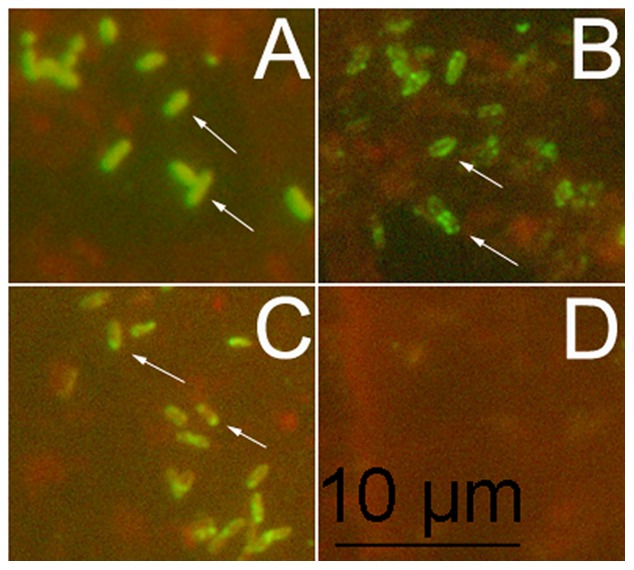
Localization of YbgF and PrsA in *R. heilongjiangensis* by immunofluorescence assay. Slides coated with *R. heilongjiangensis* cells were incubated with sera from mice immunized with *R. heilongjiangensis* whole cell antigens plus adjuvant (A), recombinant YbgF plus adjuvant (B), recombinant PrsA plus adjuvant (C), or adjuvant alone (D). After staining with Dylight 488-conjugated goat anti-mouse IgG, the rickettsial cells on the slide were observed under a fluorescence microscope (Olympus BX60).

To confirm the surface localization of the novel surface proteins, YbgF and PrsA, which were recognized in this study, *R. heilongjiangensis* cells were stained for IFAs with antibodies against YbgF or PrsA. Fluorescent rings were observed around the rickettsial cells stained with antibodies to YbgF (Figure 2B), while fluorescent spots were seen at one end of the cells stained with antibodies to PrsA (Figure 2C).

### Serological Analysis of Surface-exposed Proteins using Mouse Sera

**Figure 3:**
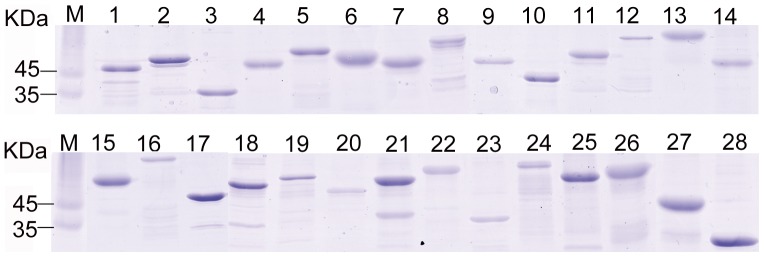
SDS-PAGE analysis of purified recombinant proteins. Twenty-four surface-exposed proteins of *R. heilongjiangensis* were successfully expressed in *E. coli* cells and purified with Ni-NTA affinity resin. Omp1, OmpA and OmpB were expressed as two, two, and three fragments, respectively. Lanes 1 to 28 refer to recombinant proteins, OmpW, RpsB, TdpX1, RfbD, CapD, PrsA, YbgF, Tuf, hypothetical protein Rh054_02285, Adr1, SurA, hypothetical protein Rh054_00610, AtpD, FolD, SdhB, DegQ, Adr2, Omp1-1, Omp1-2, OmpA-1, OmpA-2, OmpB-1, OmpB-2, OmpB-3, hypothetical protein Rh054_06655, GroEL, RplA, and RplY, respectively. Lane M refers to protein markers and their relative molecular masses are indicated in KDa on the left.

All of the SEPs identified, with Sca1 being the exception, were successfully expressed in *E. coli* cells. Omp1, OmpA and OmpB were expressed as two, two, and three fragments, respectively (Figure 3). The purified recombinant SEPs fabricated on the microarray slide were analyzed with 16 sera from *R. heilongjiangensis*-infected mice and 16 sera from normal mice (Tables 2 and S3). The average FI value of each protein probed with the sera from the infected mice was significantly higher than that probed with normal sera (p<0.05). All of the SEPs, with the exception of four proteins (i.e., Omp1-2, OmpB-1, RfbD and hypothetical protein Rh054_00610), reacted positively with at least one of the sera from the infected mice. Eleven proteins (i.e., GroEL, OmpA-2, OmpB-3, PrsA, RplA, RplY, RpsB, SdhB, SurA, YbgF and hypothetical protein Rh054_02285) reacted positively with at least half of the sera from the infected mice and were therefore considered to be the major seroreactive proteins (antigens). OmpA-2 and GroEL reacted positively with all of the sera from the infected mice and OmpA-2 had the highest average FI value (Tables 2 and S3).

**Table 2:** Average fluorescence intensity and standard deviation of each surface-exposed protein probed with the sera from *R. heilongjiangensis*-infected mice and FESF patients.

	Average fluorescence intensity ± standard deviation (positive serum No./total serum No.)			
Proteins	Infected mouse sera	Normal mouse sera	FESF patient sera	Sera of healthy people
Adr1	67.5±55 (4/16)	24.1±8.7 (0/16)	122.8±51.5 (0/9)	86.7±50.9 (0/7)
Adr2	37.9±23.8 (2/16)	16.8±10.5 (0/16)	77±29.8 (1/9)	49±33.6 (0/7)
AtpD	69.4±22.3 (6/16)	30.9±13.7 (0/16)	84.9±94.5 (0/9)	97.4±74.9 (0/7)
CapD	495.7±244.4 (3/16)	308.5±163.2 (0/16)	60.3±51 (1/9)	47.3±35.3 (0/7)
DegQ	298.5±248.1 (3/16)	104.8±168.1 (0/16)	64.7±68.1 (1/9)	58±42 (0/7)
FolD	49.9±14 (4/16)	24.5±11 (0/16)	99.5±42.8 (0/9)	89.1±70.5 (0/7)
GroEL	1085.2±606.8 (16/16)	12.9±8.2 (0/16)	522.5±879.4 (7/9)	25.4±30.3 (0/7)
Omp1-1	50.5±36.4 (1/16)	23.9±30.1 (0/16)	89.1±36.8 (2/9)	45.2±30.0 (1/7)
Omp1-2	233.4±206.5 (0/16)	195.7±154.1 (0/16)	130.6±146.2 (2/9)	73.9±43.7 (0/7)
OmpA-1	35.3±16.7 (2/16)	17.7±10.1 (0/16)	62.3±60.2 (1/9)	71.9±55 (0/7)
OmpA-2	4330.9±2886.5 (16/16)	25.6±22.9 (0/16)	1056±2055.9 (6/9)	59.7±34.9 (0/7)
OmpB-1	178.7±147.6 (0/16)	122.6±119.4 (0/16)	92.7±90.9 (1/9)	65.1±57.4 (0/7)
OmpB-2	397.6±183.7 (1/16)	248.3±166.4 (0/16)	182.6±115.1 (3/9)	118.9±56 (0/7)
OmpB-3	379.5±768.7 (13/16)	24.1±9.6 (0/16)	225.4±170.2 (3/9)	89.2±54.7 (0/7)
OmpW	340.9±295 (5/16)	123.9±147.0 (1/16)	179.4±88.1 (0/9)	142.7±79.9 (0/7)
PrsA	1630.2±2833.6 (14/16)	20.7±8.4 (0/16)	87.9±46.6 (0/9)	75.4±59.4 (1/7)
RfbD	273.4±265.1 (0/16)	252.8±232.9 (0/16)	65.7±66.5 (2/9)	38.9±44.2 (0/7)
RplA	239.9±654.5 (10/16)	22.4±9.7 (0/16)	104.3±194.1 (1/9)	37.1±31.5 (0/7)
RplY	41.4±18.9 (8/16)	20±5.2 (0/16)	106.9±37.6 (2/9)	58.3±37.7 (0/7)
RpsB	79.2±28.1 (10/16)	33±13.5 (0/16)	121.6±90.4 (3/9)	69.1±44.9 (0/7)
SdhB	82.5±27.3 (13/16)	27.1±11.1 (0/16)	198.7±343.4 (3/9)	67±41.7 (0/7)
SurA	73.4±47.7 (8/16)	21.6±14.1 (0/16)	70.9±60.3 (1/9)	53.1±40.6 (0/7)
TdpX1	359.6±216.9 (1/16)	240.9±137.5 (0/16)	82.2±84.2 (1/9)	58.4±53.9 (0/7)
Tuf	591.6±291.1 (3/16)	336±182.6 (0/16)	56±61.4 (1/9)	45.1±39.2 (0/7)
YbgF	186.8±256.8 (10/16)	24.6±11.7 (0/16)	79.6±56.3 (2/9)	32.5±24.4 (0/7)
Rh054_00610	43.7±14.8 (0/16)	23.4±14.4 (0/16)	108.2±64.8 (1/9)	87±37.5 (0/7)
Rh054_02285	182.7±200.9 (8/16)	30.2±17 (0/16)	72.3±78.9 (3/9)	49.7±49.3 (0/7)
Rh054_06655	59.7±21.4 (3/16)	30.4±13.3 (0/16)	174.7±116.3 (1/9)	133.9±105.6 (0/7)

FESF: Far-Eastern spotted fever.

### Serological Analysis of Surface-exposed Proteins using Patient Sera

The recombinant SEPs on the microarray slide were probed with nine sera from FESF patients and seven sera from healthy people. As a result (Tables 2 and S3), OmpA-2 and GroEL reacted positively against six and seven of the nine samples of patient sera, respectively; other 10 proteins reacted positively against between two and four of the nine patient serum samples, while the remaining proteins reacted with only one or none of the sera from the nine patients. Statistical analysis showed the average FI value of the GroEL protein probed with the patient sera was significantly higher than that probed with sera from healthy people (p<0.01), and that the average FI values for all of the other proteins were also higher than for those probed with sera from healthy people, but the result was not statistically significant (p>0.05).

### Specificity Analysis of Major Seroreactive Proteins using Microarray Assays

**Figure 4:**
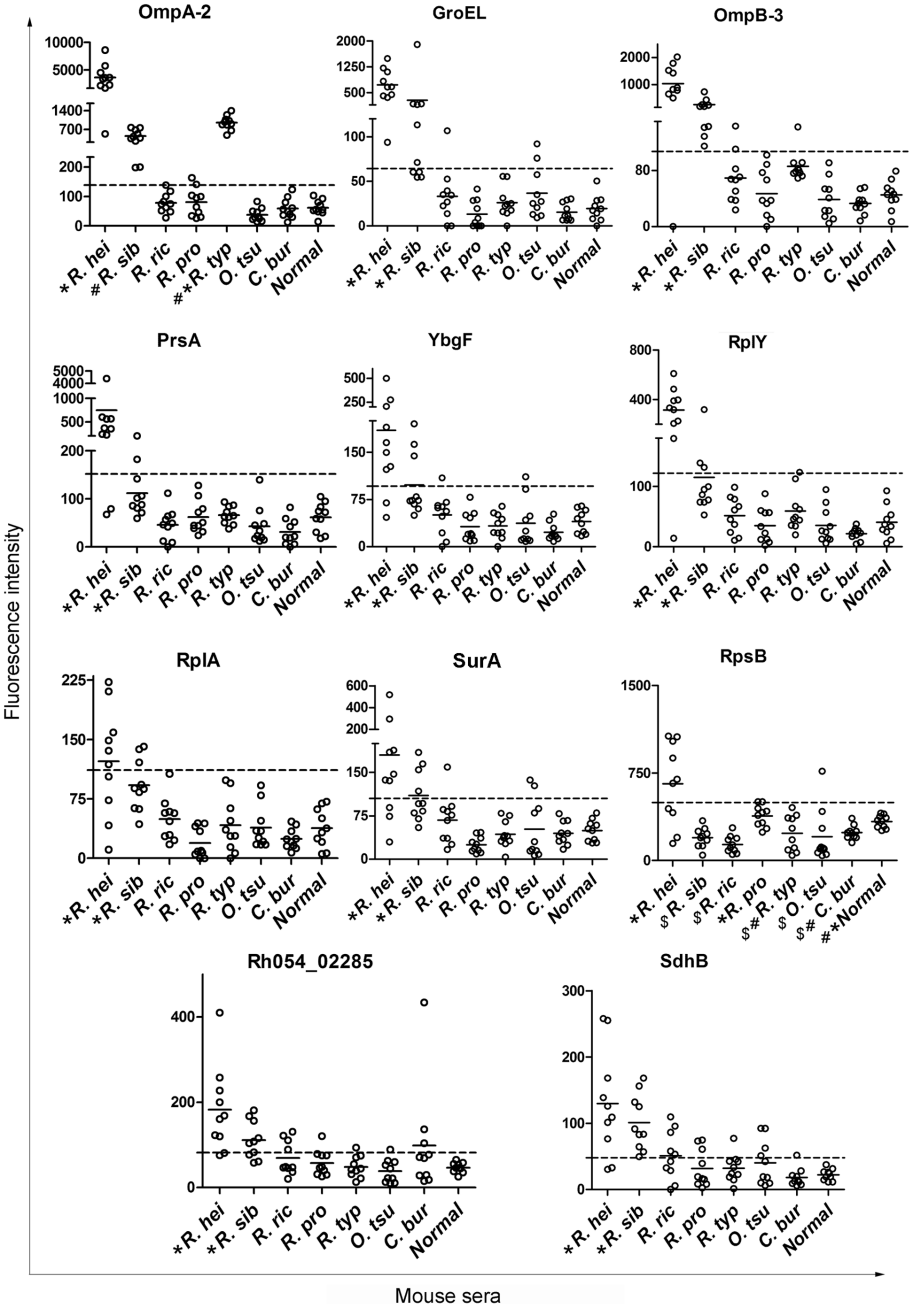
Major seroreactive surface-exposed proteins probed with the serum samples from mice infected with different rickettsia agents. Eleven major seroreactive SEPs on the microarray slide were probed with the sera from mice infected with *R. heilongjiangensis* (*R. hei*), *R. sibirica* (*R. sib*), *R. rickettsii* (*R. ric*), *R. prowazekii* (*R. pro*), *R. typhi* (*R. typ*), *O. tsutsugamushi* (*O. tsu*) or *C. burnetii* (*C. bur*), or with normal mouse sera (Normal). The figure shows the FI value distribution of the 11 proteins. The dash line on each panel reprents the average FI value plus 3 times the SD of each protein probed with normal sera, which was calculated as the cut-off to determine if the reaction was negative or positive. Short bars represent the average FI value of each group. “*”, “#” and/or “$” appear where the average FI value is significantly different from that of the other groups (p<0.05). The average FI values of the groups with the same symbols are not significantly different (p>0.05). Statistical analysis was done by the Kruskal-Wallis test, followed by the Student-Newman-Keuls (SNK) test using software SAS 9.1 (SAS Institute).

The major seroreactive SEPs on the microarray slide were probed with serum samples from mice infected with different rickettsial agents. As a result (Table 3, Figure 4 and Table S4), all of the 11 major seroreactive SEPs reacted positively with at least six of the 10 sera from *R. heilongjiangensis*-infected mice; GroEL, OmpA-2, OmpB-3, SdhB, and the hypothetical protein Rh054_02285 reacted positively with at least six of the 10 sera from *R. sibirica*-infected mice; SdhB and the hypothetical protein Rh054_02285 reacted positively with five or less of the 10 sera from mice infected with *R. rickettsii*, *R. prowazekii*, *R. typhi*, *O. tsutsugamushi* or *C. burnetii*; however, all 10 sera from the *R. typhi*-infected mice were recognized by OmpA-2.

**Table 3:** Serological specificity of the major seroreactive surface-exposed proteins of *R. heilongjiangensis* analyzed by microarray assay using serum samples from mice infected with different rickettsial agents.

	Average and standard deviation (SD) of the FI value of each protein probed with different serum samples (positive No./total No.)							
Proteins	*R. hei*	*R. sib*	*R. ric*	*R. pro*	*R. typ*	*O. tsu*	*C. bur*	Normal
GroEL	727±434.5 (10/10)	281±569.9 (6/10)	29.7±35.4 (1/10)	10.2±18.5 (0/10)	25.6±18.3 (0/10)	36.6±28.8 (2/10)	15.5±9.9 (0/10)	19.1±15 (0/10)
OmpA-2	3629.7±2294 (10/10)	464±218 (10/10)	78±33.5 (0/10)	80.6±47.9 (2/10)	953.2±258.2 (10/10)	38.0±22.0 (0/10)	58.9±33.5 (0/10)	61.9±25.6 (0/10)
OmpB-3	1038.1±629.8 (9/10)	253±189 (10/10)	69.3±36.2 (2/10)	44±40.2 (0/10)	86.1±21 (1/10)	38.9±28.9 (0/10)	33±14.8 (0/10)	45.2±20.8 (0/10)
PrsA	745.6±1296.1 (8/10)	112±48 (2/10)	45.3±34.4 (0/10)	62.1±34.0 (0/10)	66.4±18.5 (0/10)	43.1±39.2 (0/10)	30.8±27.1 (0/10)	61.8±30.1 (0/10)
RplA	122.4±68.1 (6/10)	92.1±32.5 (3/10)	49.4±26.8 (0/10)	17.7±21.3 (0/10)	40.2±36.7 (0/10)	38.7±26.8 (0/10)	24.4±12.9 (0/10)	38.1±24.4 (0/10)
RplY	316.2±169.2 (9/10)	114.9±76.5 (3/10)	51.8±30.4 (0/10)	35±28.6 (0/10)	59.4±33.9 (1/10)	35.3±29.9 (0/10)	21.3±10 (0/10)	40.6±27.1 (0/10)
RpsB	658.8±348.5 (6/10)	196.9±84.6 (0/10)	138±73.9 (0/10)	383.4±91.7 (2/10)	233±152.6 (0/10)	204.5±230.2 (1/10)	240.1±59.6 (0/10)	333.9±54.6 (0/10)
SdhB	129.6±79.6 (8/10)	101±41.8 (10/10)	51.2±37 (5/10)	31.8±28 (3/10)	32.2±21.1 (1/10)	40.4±33.3 (4/10)	18±13.7 (1/10)	22.2±8.6 (0/10)
SurA	179.9±139.2 (7/10)	110.1±43.9 (4/10)	67.6±42 (1/10)	25.1±13.7 (0/10)	43.1±23.5 (0/10)	52.2±50.9 (2/10)	44.8±20 (0/10)	49.4±18.6 (0/10)
YbgF	185±128.1 (8/10)	97.9±50.2 (3/10)	49.8±33.9 (1/10)	31.7±23.6 (0/10)	31.6±22.8 (0/10)	37.1±36.9 (1/10)	23±13.9 (0/10)	39.8±18.9 (0/10)
Rh054_02285	182.5±99.9 (8/10)	111.3±44.1 (7/10)	69.8±39.6 (4/10)	57.3±29.8 (1/10)	48.6±25.1 (1/10)	38.7±27.1 (1/10)	98.5±124.3 (3/10)	46.8±11.7 (0/10)

The major seroreactive surface-exposed proteins of *R. heilongjiangensis* fabricated on a microarray slide were probed with the sera from mice infected with *R. heilongjiangensis* (*R. hei*), *R. sibirica* (*R. sib*), *R. rickettsii* (*R. ric*), *R. prowazekii* (*R. pro*), *R. typhi* (*R. typ*), *O. tsutsugamushi* (*O. tsu*) or *C. burnetii* (*C. bur*), or normal mouse sera (Normal).

The average FI values of the major seroreactive SEPs (but not RpsB and OmpA-2) probed with the sera from mice infected with *R. heilongjiangensis* or *R. sibirica* were significantly higher than those probed with sera from mice infected with any of the other rickettsial bacteria (p<0.05) (Figure 4). The average FI value of OmpA-2 probed with sera from *R. heilongjiangensis*-infected mice was not significantly higher than that probed with the sera from *R. typhi*-infected mice (p>0.05), but was significantly higher than that probed with sera from mice infected with any of the other rickettsial bacteria (p<0.05) (Figure 4).

### Specificity Analysis of Major Seroreactive Proteins using ELISA

The specificity of the major seroreactive SEPs was also analyzed by ELISA using sera from mice that had been infected with the different rickettsial bacteria. As shown in Table 4, all of the SEPs (except SdhB and Rh054_02285) reacted positively with between five and 10 of the 10 serum samples from the mice infected with *R. heilongjiangensis*; all of the SEPs reacted positively with at least six out of 10 sera from mice infected with *R. sibirica* or *R. rickettsii*, and with between four and six of the 10 sera from mice infected with *O. tsutsugamushi*. We also found that only one to three out of 10 sera from mice infected with *R. prowazekii* were recognized by some of the SEPs and only one out of 10 sera from mice infected with *R. typhi* or *C. burnetii* were recognized by the SEPs, the exceptions being OmpB-3 and RplY. The concordance of the seroreactivity of the SEPs between the ELISA and microarray assay results was 75% (Table 5).

**Table 4:** Serological specificity of the major seroreactive surface-exposed proteins of *R. heilongjiangensis* analyzed by ELISA using serum samples from mice infected with different rickettsial agents.

	Average and standard deviation (SD) of the optical density of each protein from ELISAs using various sera (positive No./total No.)							
Proteins	*R. hei*	*R. sib*	*R. ric*	*R. pro*	*R. typ*	*O. tsu*	*C. bur*	Normal
GroEL	1.31±0.37 (10/10)	0.6±0.47 (10/10)	0.75±0.43 (10/10)	0.13±0.02 (1/10)	0.14±0.02 (0/10)	0.36±0.38 (5/10)	0.14±0.02 (1/10)	0.11±0.01 (0/10)
OmpA-2	1.83±0.23 (10/10)	0.73±0.2 (10/10)	0.44±0.29 (8/10)	0.14±0.03 (0/10)	0.15±0.02 (0/10)	0.28±0.22 (4/10)	0.15±0.02 (0/10)	0.14±0.03 (0/10)
OmpB-3	1.01±0.59 (10/10)	0.35±0.29 (8/10)	0.31±0.11 (7/10)	0.17±0.05 (1/10)	0.23±0.04 (6/10)	0.24±0.14 (5/10)	0.18±0.04 (2/10)	0.14±0.02 (0/10)
PrsA	0.85±0.76 (8/10)	0.28±0.08 (8/10)	0.29±0.09 (8/10)	0.16±0.06 (1/10)	0.16±0.03 (0/10)	0.32±0.31 (5/10)	0.13±0.02 (0/10)	0.13±0.02 (0/10)
RplA	0.22±0.07 (5/10)	0.29±0.1 (8/10)	0.27±0.13 (6/10)	0.17±0.06 (3/10)	0.16±0.04 (1/10)	0.19±0.08 (4/10)	0.15±0.02 (0/10)	0.13±0.02 (0/10)
RplY	0.32±0.16 (7/10)	0.54±0.15 (10/10)	0.52±0.31 (7/10)	0.19±0.05 (2/10)	0.22±0.04 (4/10)	0.38±0.26 (6/10)	0.23±0.07 (4/10)	0.14±0.02 (0/10)
RpsB	0.3±0.07 (6/10)	0.43±0.11 (9/10)	0.37±0.18 (6/10)	0.22±0.07 (2/10)	0.21±0.04 (1/10)	0.24±0.13 (4/10)	0.17±0.02 (0/10)	0.15±0.03 (0/10)
SdhB	0.21±0.04 (3/10)	0.38±0.1 (9/10)	0.34±0.15 (6/10)	0.17±0.05 (1/10)	0.19±0.03 (1/10)	0.27±0.17 (5/10)	0.15±0.02 (0/10)	0.14±0.02 (0/10)
SurA	0.27±0.05 (6/10)	0.4±0.1 (10/10)	0.36±0.2 (6/10)	0.25±0.19 (3/10)	0.17±0.04 (0/10)	0.28±0.16 (6/10)	0.17±0.04 (0/10)	0.16±0.03 (0/10)
YbgF	0.25±0.05 (8/10)	0.29±0.07 (9/10)	0.44±0.41 (7/10)	0.25±0.14 (3/10)	0.18±0.04 (1/10)	0.4±0.47 (5/10)	0.15±0.03 (0/10)	0.14±0.02 (0/10)
Rh054_02285	0.22±0.04 (2/10)	0.4±0.11 (10/10)	0.31±0.14 (6/10)	0.23±0.1 (3/10)	0.19±0.04 (1/10)	0.31±0.21 (5/10)	0.15±0.02 (0/10)	0.15±0.02 (0/10)

The sera from mice infected with *R. heilongjiangensis (R. hei), R. sibirica (R. sib), R. rickettsii (R. ric), R. prowazekii (R. pro), R. typhi (R. typ), O. tsutsugamushi (O. tsu) or C. burnetii (C. bur)*, or normal mouse sera (Normal) were used to test the specificity of the major seroreactive surface-exposed proteins of *R. heilongjiangensis*.

**Table 5:** Serological sensitivity and specificity values of the major seroreactive surface-exposed proteins of *R. heilongjiangensis* from microarray and ELISA tests.

	Microarray assay					ELISA					
	*R. hei*		SFG			*R. hei*		SFG			
Proteins	Se	Sp	Se	Sp	Remarks*	Se	Sp	Se	Sp	Remarks*	Concordance#
GroEL	100	87	57	96	Marker *R. hei*	100	61	100	86	Marker SFG	78%
OmpA-2	100	69	67	76		100	69	93	92	Marker SFG	70%
OmpB-3	90	81	70	98	Marker SFG	100	59	83	72		74%
PrsA	80	97	33	100	Marker *R. hei*	80	69	80	88	Marker SFG	75%
RplA	60	96	30	100		50	69	63	84		75%
RplY	90	94	40	98	Marker *R. hei*	70	53	80	68		61%
RpsB	60	96	20	94		60	69	70	86	Marker SFG	76%
SdhB	80	66	77	82		30	69	60	86		86%
SurA	70	90	40	96	Marker *R. hei*	60	64	73	82		76%
YbgF	80	93	40	98	Marker *R. hei*	80	64	80	82		75%
Rh054_02285	80	76	63	88		20	64	60	82		75%

*R. hei*, *R. heilongjiangensis*; SFG, Spotted fever group; Se, sensitivity; Sp, specificity.

* proteins with >70% sensitivity and >85% specificity were selected as potential markers for *R. heilongjiangensis* or SFG rickettsia infections.

# concordance between ELISA and microarray assays used in the present study.

## Discussion

The SEPs of obligate intracellular bacteria provide a crucial interface for interactions between bacteria and host cells [40]. SEPs mediate the initial attachment of a bacterium to a host cell and subsequent contact with host cytosolic proteins, a process that promotes bacterial survival and replication by subverting host cellular processes [40]. Definition of *R. heilongjiangensis* SEPs will provide an important starting point towards better understanding of the interactions that take place at the interface of rickettsia and host cells. In the present study, we identified 25 *R. heilongjiangensis* SEPs using biotin-streptavidin affinity chromatography coupled with 2D-PAGE and ESI-MS/MS.

Some of the proteins were present in more than one spot in the gel, with the maximum number being 11 spots per protein. The theoretical molecular weights and/or pI values of the proteins did not correlate with the values obtained from the experiments. This may have been caused by the Sulfo-NHS-SS-Biotin residues that were not eluted during purification as well as post-translational modifications of rickettsial proteins [38], [41]. OmpB was identified in eight spots (spots 13, 15, 18, 19, 22–24, and 48). Only spot 48 matched its theoretical size and pI; therefore, the other seven spots with molecular masses of ∼35 kDa were considered to be its β-peptide. Where proteins were identified in several spots in the gel, they were more likely to be highly abundant *R. heilongjiangensis* cell surface proteins.

In the present study, nine proteins, including Adr1, Adr2, OmpA, OmpB, OmpW, PrsA, Sca1, YbgF, and the hypothetical protein Rh054_00610, were predicted to have both classical and non-classical secretion signals by the SignalP/LipoP and SecretomeP bioinformatics programs. Adr1 and Adr2 have been identified as important rickettsial adhesins [37], [42], [43]. Previous studies showed that antibodies to Adr1 in *R. conorii* and Adr2 in *R. prowazekii* inhibited entry of the homologous bacteria into host cells, suggesting they perform invasion-related roles [42], [43]. OmpA (Sca0), OmpB (Sca5), and Sca1 are involved in rickettsial attachment and/or entry to host cells [13]–[15], [17], [18]. While PrsA is an extracellular chaperone and membrane-bound lipoprotein that is essential for growth and protein secretion in *Bacillus* [44], [45], YbgF is a periplasmic protein belonging to the Tol-Pal system, which maintains cell envelope integrity and also serves a role in the import of virulence factors of pathogenic bacteria [46]. In the present study, IFAs using antibodies against YbgF and PrsA revealed the presence of fluorescent rings and spots around the *R. heilongjiangensis* cells and at one end of the cells, respectively, suggesting they are localized on the surface of the bacteria (Figure 2). However, whether they are periplasmic or membrane-bound proteins requires further investigation.

Roles for OmpW and hypothetical protein Rh054_00610 in bacterial pathogenesis have not been demonstrated. Four proteins, DegQ, Omp1, SurA, and hypothetical protein Rh054_02285, have classical secretion signal peptides, while hypothetical protein Rh054_06655 has a non-classical secretion signal. DegQ is a periplasmic serine protease, which is not essential for bacterial pathogenesis, but may play a small role during *salmonella* growth at systemic sites [47]. SurA is either a periplasmic prolyl isomerase or chaperone that facilitates outer membrane protein biogenesis and pilus assembly in *E. coli* [48]. Hypothetical protein Rh054_02285 was identified as a haloacid dehalogenase-like hydrolase. *R. heilongjiangensis* homologues of these secretion proteins (with the exceptions of SurA and the hypothetical protein Rh054_02285) reside on the surface of *R. conorii* [15], [36], [37], *R. felis* [38], *R. parkeri* [39], *R. typhi* [40], *Anaplasma phagocytophilum* [24], and/or *Ehrlichia chaffeensis* [25] (Table 1). In addition, the classical secretion proteins in *R. heilongjiangensis* (except DegQ) are Sec-dependent extracytoplasmic proteins in *R. typhi* [49].

The remaining 11 SEPs were not predicted to have secretion signals and are usually considered to be cytosolic proteins. However, proteins with homology to AtpD, GroEL, RpsB, TdpX1, and Tuf of *R. heilongjiangensis* were found on the cell surfaces or in the membrane fractions of *R. conorii* [36], [37], *R. felis* [38], *R. parkeri* [39], *R. typhi* [40] and/or other Gram-negative bacteria [24], [25], [41] (Table 1).

AtpD is the inner membrane-localized β-subunit of an ATPase involved in bacterial adenosine nucleotide *de novo* biosynthesis [50]. GroEL is a heat shock protein that has been shown to be located on the surfaces of other rickettsial bacteria [24], [25], [40]. TdpX1 (thioredoxin peroxidase 1) defends against oxidant stress in *Leishmania* [51] and has been found in rickettsial organisms but not in free-living bacteria [52], indicating that its presence on the surface of rickettsial cells may afford protection against the oxidant stress encountered inside host cells. Tuf has been recognized as an adhesin-like factor with the ability to mediate attachment of *Lactobacillus johnsonii* to intestinal epithelial cells and mucins [53].

Three ribosomal proteins, L1 (RplA), L25 (RplY) and S2 (RpsB), were identified among the secretion signal-lacking SEPs in the present study. RpsB is found on the surface of *R. felis* [38] and in the present study is predicted to be an extracellular protein by SOSUI-GramN (Table 1). Indeed, some ribosomal proteins have been recognized as membrane-associated proteins in bacteria [38], [39], while the surface proteins L12 [54] and L25 [55] have been shown to be involved in bacterial pathogenicity [54], [56].

Four other proteins, CapD, RfbD, SdhB, and FolD, were identified on the surface of *R. heilongjiangensis* cells in the present study. CapD, a UDP-glucose 4-epimerase [57], and RfbD, a dTDP-4-dehydroamnose reductase, both contribute to exopolysaccharide synthesis in Gram-negative bacteria [57], [58]. SdhB and FolD are succinate dehydrogenase iron-sulfur subunit and methylenetetrahydrofolate dehydrogenase or cyclohydrolase, respectively, and they may be associated with synthesis of membrane carbohydrates or energy metabolism in bacterial membranes. In previous studies, cytosol aminopeptidase has been found in the outer membrane fraction of *A. marginale* [59], while disulfide oxidoreductase has been observed on the bacterial surface of *E*. *chaffeensis* [60], and elongation factor G and the ATP synthase F1 alpha and beta subunits have been detected on the cell envelope of *Staphylococcus aureus* [61]. Therefore, these proteins with well-known functions in bacterial membranes may also be present on the surface of *Rickettsiae* and play unexpected roles in rickettsia-host interactions.

To further characterize their roles in the pathogenicity and immunogenicity of *R. heilongjiangensis*, the recombinant SEPs were used to fabricate a microarray for analysis with sera from *R. heilongjiangensis*-infected mice and FESF patients. Eleven SEPs, including GroEL, OmpA-2, OmpB-3, PrsA, RplA, RplY, RpsB, SdhB, SurA, YbgF, and hypothetical protein Rh054_02285, were recognized as major seroreactive antigens by the sera from *R. heilongjiangensis*-infected mice. OmpA and OmpB were expressed as two or three fragments; however, only the C-terminal fragments (OmpA-2 and OmpB-3) reacted strongly with these sera, which could be related to the presence of a conserved outer membrane autotransporter domain located at the C-terminal fragments of both proteins.

In the present study, GroEL and PrsA were identified in 10 and 13 spots on the 2D-PAGE gel, respectively, suggesting that they are both highly abundant surface proteins in *R. heilongjiangensis*. YbgF is recognized as a fairly abundant seroreactive protein, which was identified in several spots in the immunoproteomic assay of *C. burnetii* [62] and has been suggested as a serological marker for Q fever [26].

In our microarray analysis, OmpA-2 and GroEL showed the strongest seroreactivity with the sera from *R. heilongjiangensis*-infected mice and FESF patients, indicating that they may be attractive candidates for the development of serodiagnostic tests for FESF.

The other major seroreactive SEPs that were identified using sera from *R. heilongjiangensis*-infected mice were poorly recognized by the FESF patient sera, suggesting that the immune profile of these SEPs in mice experimentally infected with *R. heilongjiangensis* differed markedly from that in patients that were naturally infected with this pathogen. However, some of the SEPs, including OmpB-3, RpsB, SdhB and hypothetical protein Rh054_02285, still reacted positively with 33% of the serum samples from FESF patients. This indicates that combining these SEPs with OmpA-2 and GroEL may lead to significant improvements in sensitivity and specificity for serodiagnosis of FESF.

To explore their serological specificity, the major seroreactive *R. heilongjiangensis* SEPs were analyzed by microarray and ELISA using sera from mice infected with different rickettsial agents. Eleven, five and one of the 11 major seroreactive SEPs were recognized by more than 50% of the sera in the microarray assays from mice infected with *R. heilongjiangensis*, *R. sibirica* or *R. rickettsii*, respectively. The average FI values of the 11 SEPs probed with the sera from *R. sibirica*- or *R. rickettsii*-infected mice were lower or significantly lower than those probed with the sera from *R. heilongjiangensis*-infected mice (Figure 4). This result is consistent with the phylogenetic analysis of SFG rickettsiae, which shows that *R. heilongjiangensis* is much more closely related to *R. sibirica* than *R. rickettsii* [1].

In addition, all the major seroreactive SEPs, with the exception of OmpA-2, SdhB, and hypothetical protein Rh054_02285, reacted positively with few of the sera from mice infected with the non-SFG rickettsiae (Figure 4). The microarray results suggest that GroEL, PrsA, RplY, SurA, and YbgF may be potential markers of *R. heilongjiangensis* infection and OmpB-3 may be a potential marker of SFG rickettsia infection since they had over 70% sensitivity and 85% specificity in this serological analysis (Table 5).

The ELISA test showed that most of the major seroreactive SEPs had higher seroreactivity to the sera from mice infected with *R. sibirica*, *R. rickettsii*, *R. prowazekii*, or *O. tsutsugamushi* compared with the microarray assay. The higher seroreactivity of these proteins might be caused by denaturation in the ELISAs [63]. It is possible that the denatured proteins may have provided more epitopes that could cross-react with the various samples of sera tested here and that the proteins on the microarray slide may have maintained their native structures and activities [63]. Some proteins, such as OmpB-3 and RplY, exhibited high cross-reactivity with some of the sera from mice infected with non-SFG rickettsiae (Table 4). This might be related to the highly conserved amino acid structures of these proteins in rickettsiae. OmpB is recognized as having high sequence conservation in rickettsiae [16]–[18], while RplY also exhibits high amino acid sequence conservation as shown by sequence alignments of the seven rickettsial species used in the present study (Table S5). Interestingly, OmpA-2 reacted positively in the microarray assay with all 10 sera from *R. typhi*-infected mice, but with none of these sera in ELISA. The tertiary structure of OmpA-2 on the microarray slide may have exerted a steric effect that promoted non-specific absorption of IgG from the sera, an effect that would not apply to the denatured OmpA-2 in the ELISAs. This, combined with the small number of serum samples, may have affected concordance between the two assays. As shown in Table 5, GroEL, OmpA-2, PrsA, and RpsB had a high sensitivity (>70%) and specificity (>85%) for SFG rickettsia (*R. heilongjiangensis*, *R. sibirica*, and *R. rickettsii*) antibodies. However, the cross-reactivity of these proteins against *O. tsutsugamushi* antibodies requires further investigation.

Many studies have shown that OmpA and OmpB elicit protective immune responses against rickettsiosis in laboratory animals [22], [64]–[66], but the immunoprotectivity of the remaining major seroreactive SEPs of *R. heilongjiangensis* merits further investigation.

In the present study, 25 *R. heilongjiangensis* SEPs were identified using biotin-streptavidin affinity purification and 2D electrophoreses coupled with ESI-MS/MS. Most of these proteins were predicted to reside on the bacterial cell surface and play roles in bacterial pathogenesis. The recombinant SEPs fabricated on the microarray slide were probed with sera from mice infected with different rickettsial agents and 11 of them were recognized as major seroreactive antigens. Among the major seroreactive SEPs, GroEL, OmpA-2, OmpB-3, PrsA, RplY, RpsB, SurA and YbgF exhibited modest sensitivity and specificity for recognizing *R. heilongjiangensis* infection and/or spotted fever in the protein microarray assay and/or the ELISAs.

Our results suggest that most of the *R. heilongjiangensis* SEPs have potential roles in bacterial pathogenicity and that some of them could become candidate molecules for serodiagnosis of and subunit vaccines antigens against FESF.

## Supporting Information

Table S1 **Primer pairs designed to amplify genes encoding the surface-exposed proteins of** ***Rickettsia heilongjiangensis*** **.**
(XLS)

Table S2 **Surface-exposed proteins of** ***Rickettsia heilongjiangensis*** **identified by ESI-MS/MS.**
(XLS)

Table S3 **Average fluorescence intensity of each surface-exposed protein probed with** ***Rickettsia heilongjiangensis*** **-infected mouse sera or FESF patient sera.**
(XLS)

Table S4 **Average fluorescence intensity of each major seroreactive surface-exposed protein probed with serum samples from mice infected with different rickettsial agents.**
(XLS)

Table S5 **BLASTP amino acid sequence alignment of** ***R. heilongjiangensis*** **RplY and homologous proteins from other rickettsial agents.**
(XLS)
